# The multidimensional needs of chronic heart failure patients and caregivers from a dyadic perspective: a scoping review

**DOI:** 10.1007/s10741-026-10616-4

**Published:** 2026-03-27

**Authors:** Cosimo Chelazzi, Daniele Marelli, Carla Ida Ripamonti, Matteo Pagnesi, Marco Metra, Philip Larkin, Geert-Jan Geersing, Carlo Leget, Klaus K. Witte, Everlien de Graaf

**Affiliations:** 1 Department of Medical and Surgical Specialties, Radiological Sciences, and Public Health, Università di Brescia, Brescia, Italy; 2 SC Cure Palliative e ADI, ASST Spedali Civili di Brescia, Brescia, Italy; 3 Institute of Cardiology, ASST Spedali Civili, University of Brescia, Brescia, Italy; 4 Cardiology, Vita-Salute San Raffaele University, IRCCS San Raffaele Hospital, Milan, Italy; 5 Lausanne University Hospital and University of Lausanne, Lausanne, Switzerland; 6 Julius Center for Health Sciences and Primary Care – Dept. of General Practice and Nursing Science, Universiteit Medisch Centrum Utrecht, Utrecht University, Utrecht, The Netherlands; 7 Department of Care Ethics, University for Humanistic Studies, Utrecht, The Netherlands; 8 University of Leeds, Leeds, UK

**Keywords:** Heart failure, Cardiology, Palliative care, Patients, Informal caregivers, Dyadic, Europe

## Abstract

Despite advances in medical and device management, people with chronic heart failure continue to experience persistent physical and non-physical symptoms. Consequently, heart failure guidelines advocate for the early integration of palliative care. Nevertheless, the multidimensional needs of patients and their informal caregivers remain insufficiently examined. This scoping review aimed to explore the physical and non-physical needs of adults living with chronic heart failure, along with those of their informal caregivers, across Europe. Following Arksey and O’Malley’s framework, a systematic search of PubMed, EMBASE, PsycINFO, CINAHL, Cochrane Library, and Web of Science was undertaken. Eligible studies, published January 2000-January 2024, reported on the multidimensional needs of adult patients and their informal caregivers in Europe. Findings were meta-aggregated to identify dyad-level mechanisms. Across the 34 included studies, involving 886 patients and 525 caregivers, both groups reported several multidimensional burdens. Patients commonly experienced physical symptoms, alongside significant psychological, social-financial, and spiritual/existential concerns. Informal caregivers faced considerable physical, emotional, and socio-financial strain, with their quality of life closely linked to the patient’s condition. Although most studies were qualitative and did not formally analyse dyadic interdependence, co-occurrence across reports allowed identification of shared dyad-level concerns, including shared prognostic uncertainty, role renegotiation, system navigation burden, and existential adaptation. Mapping these issues supports a needs-based rationale for earlier and more structured integration of palliative care within routine chronic heart failure management.

**Supplementary information:**

The online version contains supplementary material available at 10.1007/s10741-026-10616-4.

## Introduction

Chronic heart failure (HF) is a major contributor to global morbidity and mortality [1]. Despite advances in disease-modifying therapies, many patients continue to experience persistent symptoms, including breathlessness, fluid retention, pain, drowsiness, and fatigue [2–10]. Beyond the physical domain, patients and their informal caregivers (IC) frequently face non-physical issues that further impair functioning and overall quality of life (QoL) [11–25]. This multidimensional burden is particularly pronounced in, but not limited to, advanced HF, where patients endure a complex interplay of refractory symptoms and non-physical distress, while IC may face significant consequences for their own QoL [26–29]. As a result, the care for people with chronic HF must extend beyond symptom control and proactively incorporate support for IC [30–32].

Recognition of these broader needs has contributed to greater interest in earlier symptom management and supportive approaches in chronic HF [33–36]. The concept of “total pain”, introduced by Cicely Saunders more than sixty years ago, offers a valuable framework for understanding the multidimensional suffering associated with life-limiting illnesses, integrating physical, psychological, social, financial, and spiritual aspects [37]. This framework forms the foundation of palliative care (PC), a holistic approach designed to address the full spectrum of needs experienced by individuals with life-limiting conditions [38]. PC is increasingly acknowledged as an essential component of comprehensive chronic HF management [1, 39, 40], with early integration associated with improved symptom control and QoL for both patients and IC, increased advance care planning (ACP), reductions in hospital readmissions, and, in some settings, lower healthcare expenditure [33, 34].

Despite these potential benefits, incorporating PC into routine chronic HF care remains challenging. Among the key barriers are the limited understanding of the multidimensional needs of patients and how best to assess and address them. Moreover, the needs of the informal caregivers are easily overlooked within a traditional disease-oriented clinical approach, despite their crucial role in providing assistance to patients living with chronic HF.

PC involvement may help those needs being assessed and addressed, contributing to overall dyadic well being and QoL. This also implies a deep knowledge of informal caregivers needs, which are less well explored as those of patients. This gap is particularly evident within the European context [41, 42].

To address these issues, this scoping review aims to map the physical, psychological, social-financial, and existential/spiritual needs and concerns of both patients living with chronic HF, irrespective of disease stage, and their IC reported in European literature.

The purpose of gathering this knowledge is to promote the development and implementation of effective early PC interventions for this population. This review is part of the European project “integRAting a Palliative care approacH for pAtients with Advanced hEart failure” (RAPHAEL), which seeks to implement a model of early PC integration into the care of people with chronic HF.

## Methods

This scoping review followed the six-stage framework described by Arksey and O’Malley and expanded by Levac and colleagues [43, 44].The scoping review format was chosen due to the broad nature of the domain of suffering and the expected heterogeneity of methods and results used to gather evidence on the nature of needs. The results are reported in accordance with the Preferred Reporting Items for Systematic Reviews and Meta-Analyses for Scoping Reviews (PRISMA-ScR) guidelines [45]. A detailed description of the methodology is provided in Supplement 1.

### Search strategy and study selection

A systematic search was conducted in EMBASE, MEDLINE, PsycINFO, CINAHL, Cochrane Library, and Web of Science. The search strategy combined terms describing the population (e.g., “chronic heart failure”) and the concepts of interest (e.g., “physical symptoms,” “psychological needs,” “social needs”). Boolean operators were used to refine search terms for Medline (Ovid SP), which was adapted for different databases (see Supplement 2). All retrieved records were uploaded to the Rayyan software for deduplication and a two-stage screening (see Supplement 1) [46].

Only European peer-reviewed studies published in English between January 2000 and January 2024, reporting directly on the multidimensional needs of patients with chronic HF and their caregivers, were included. Studies conducted outside Europe, publications not in English, and grey literature (including conference abstracts, editorials, letters, and non-indexed reports) were excluded (see Supplementary Material 1 for detailed inclusion and exclusion criteria).

### Data charting and synthesis

As is standard for scoping reviews, a formal methodological quality appraisal of the included studies was not performed. Data extracted included: publication details (first author, country), study characteristics (design, sample size), and key findings related to the multidimensional needs and concerns of both patients and their caregivers. Additionally, studies were classified based on their data source configuration: true dyadic studies (paired patient–caregiver data); studies reporting on both patients and caregivers, but unpaired; patient-only studies; caregiver-only studies.

The extracted data were synthesized descriptively to identify and summarize the key needs reported across the literature. To allow for the interpretation of needs as “dyadic burdens”, findings were meta-aggregated to identify dyadic-level themes. Finally, the synthesized results were shared and discussed with consortium members, including patient representatives and healthcare professionals, to incorporate their perspectives and refine the conclusions. To underline the dyadic perspective of living with chronic HF, the results are presented from both perspectives per domain of suffering, building on the conceptual construct of “total pain” [33].

## Results

### Overview

**Fig. 1:**
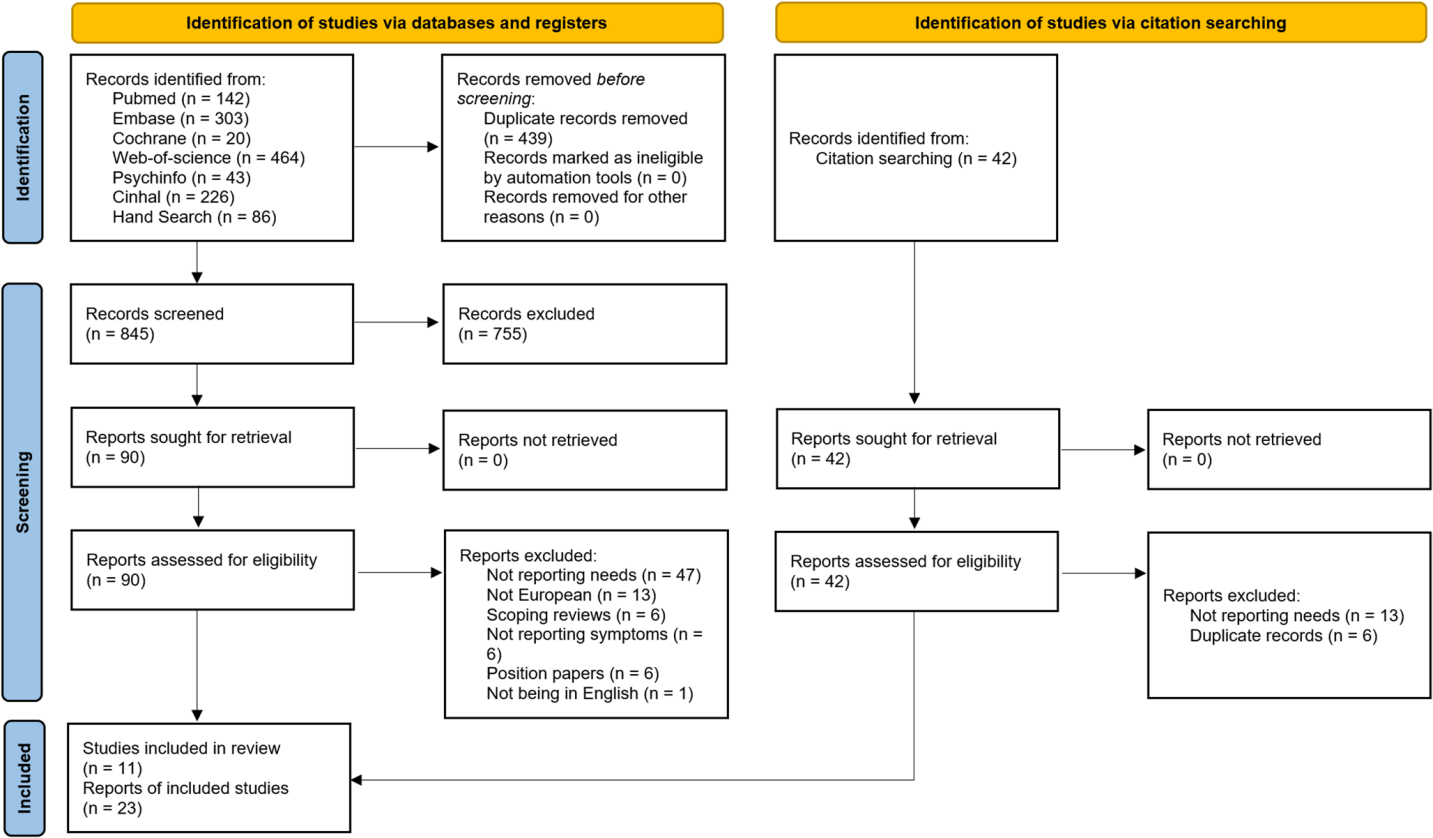
PRISMA Flow Diagram for the scoping review process

A total of 1,198 records were identified from electronic databases (MEDLINE, 142; Embase, 303; Cochrane, 20; Web of Science, 464; PsycINFO, 43; CINAHL, 226), and additional 86 records were retrieved through hand searching (see Fig. 1). After removal of 439 duplicates, 845 abstracts were screened, of which 755 were excluded.

Following abstract screening, 90 full texts were assessed, of which 11 papers were included in the analysis, and 79 were excluded for: not directly reporting needs (*n* = 47); not being European/not reporting European data (*n* = 13); not reporting symptoms (*n* = 6); being scoping or position papers (*n* = 12); not being in English (*n* = 1).

Subsequently, citation searching identified 42 additional records, of which 23 met the inclusion criteria. Detailed reasons for exclusion after screening are presented in the PRISMA-ScR flow diagram (Fig. 1).

Finally, 34 papers were included for data extraction, collectively involving 886 patients and 525 caregivers (see Table 1).

**Table 1:** Description of included studies

1 st Author et al. (Ref)	Title	Design of study	Country of origin	Sample size	Reporting pattern	HF Phenotype	Patient’s symptoms, needs, or concerns	Caregivers needs or concerns
Andersson L et al. [47]	Living with heart failure without realising: a qualitative patient study	Qualitative patient study	Sweden	11 Patients	Patients only	Not reported	Continuity of care Communication/information Fatigue, breathlessness. Being in control/autonomous	
Boyd KJ et al. [48]	Living with advanced heart failure: a prospective, community based study of patients and their carers	Prospective longitudinal study	United Kingdom	20 Patients Undisclosed Caregivers	Both (not paired	Not reported	Breathlessness/Dyspnoea, Oedema burden, Fatigue, Muscle issues/weakness, Poor appetite/Anorexia, Sleep disorders Anxiety, Depression, Frustration Advance care planning, sense of isolation, continuity of care, communication/information Being autonomous or in control, Hope, end of life care, having meaning	Emotional strain/burden Changes in dyad roles/family issues, Social isolation, social and financial burden of care
Brännström M et al. [49]	Being a close relative of a person with severe, chronic heart failure in palliative advanced home care – a comfort but also a strain	Qualitative study	Sweden	4 Caregivers	Caregivers only	Not reported		Physical burden of caring 24/7 Emotional strain/burden Care Continuity, Social isolation Feeling supported
Browne S et al. [50]	Patient, Carer and Professional Perspectives on Barriers and Facilitators to Quality Care in Advanced Heart Failure	Qualitative study	United Kingdom	30 Patients 20 Caregivers	Both (not paired)	Not reported	Communication/information Referral to Palliative Care, Advance care planning, Being connected, Continuity of care, Understanding of treatments and devices Awareness of dying, Being autonomous or in control	Care continuity, Lack of services, Limited or lack of communication with HCPs, Social and financial burden of care, Understanding of treatments and devices
Chester R et al. [51]	Heart failure—the experience of living with end-stage heart failure and accessing care across settings	Qualitative study	United Kingdom	4 Patients 4 Caregivers	True dyadic (paired)	Not reported	Advance care planning, Continuity of care, Communication/information Referral to Palliative Care, Understanding of treatments and devices	Care continuity, Limited or lack of communication with HCPs, Understanding of treatments and devices Limited/inadequate end-of-life and bereavement support
Cortis JD et al. [52]	Palliative and supportive needs of older adults with heart failure	Qualitative study	United Kingdom	10 Patients	Patients only	Not reported	Breathlessness/dyspnea, Chest discomfort/chest pain, Fatigue, Falls, Oedema burden, Poor appetite/Anorexia, Sleep disorders Anxiety, Fear, Frustration, Self-esteem Communication/information, Sense of isolation Understanding prognosis, Understanding of treatments and devices Being autonomous or in control, Spiritual support	
Durante A et al. [53]	Needs and problems related to sociodemographic factors of informal caregiving of people with heart failure: A mixed methods study in three European countries	Qualitative mixed methods study	Italy, Spain, The Netherlands	52 Caregivers	Caregivers only	Not reported		Physical burden of caring 24/7 Emotional strain/burden, Depression, Changes in dyad roles/family issues, Limited or lack of communication with HCPs, Social isolation, Social support Unpreparedness/fear for the future
Durante A et al. [54]	Informal caregivers of people with heart failure and resilience: A convergent mixed methods study	Qualitative study	Italy, Spain, The Netherlands	50 Caregivers	Caregivers only	Not reported		Physical burden of caring 24/7, Physical health Anxiety, Depression, Emotional strain/burden, Engaging in self comforting activities Care continuity, Changes in dyad roles/family issues, Lifestyle changes, Limited or lack of communication with HCPs, Social support, Social and financial burden of care Hopelessness
Falk S et al. [55]	Keeping the maintenance of daily life in spite of Chronic Heart Failure. A qualitative study	Qualitative study	Sweden	17 Patients	Patients only	Not reported	Being connected, Continuity of care, Understanding of treatments and devices Awareness of dying, Being autonomous or in control, Having meaning, Illness perception	
Finamore P et al. [56]	Clustering of patients with end-stage chronic diseases by symptoms: a new approach to identify health needs	Observational prospective study	The Netherlands	80 Patients	Patients only	Not reported	Chest discomfort/chest pain, Cough, Dizziness/drowsiness, Breathlessness/dyspnoea, Fatigue, Mouth issues, Muscle issues/weakness, Oedema burden, Pain, Poor appetite/Anorexia, Pruritus, Restless leg syndrome, Sleep disorders, Thirst Anxiety, Depression	
Fitzsimons D et al. [57]	Inadequate Communication Exacerbates the Support Needs of Current and Bereaved Caregivers in Advanced Heart Failure and Impedes Shared Decision-making	Qualitative study	Ireland, United Kingdom	30 Caregivers (20 current and 10 bereaved)	Caregivers only	HFrEF (Ejection fraction < 40%)		Physical burden of caring 24/7 Emotional strain/burden, Understanding of Palliative Care, Care continuity, Lack of services, Limited or lack of communication with HCPs, Understanding of treatments and devices Limited/inadequate end-of-life and bereavement support
Fry M et al. [58]	The implications of living with heart failure; the impact on everyday life, family support, co-morbidities and access to healthcare: a secondary qualitative analysis	Secondary qualitative analysis	United Kingdom	11 Patients	Patients only	Not reported	Oedema burden Concerns about comorbidities, Being connected, Communication/informationContinuity of care, Family concerns	
Gallacher K et al. [59]	Understanding Patients’ Experiences of Treatment Burden in Chronic Heart Failure Using Normalization Process Theory	Secondary qualitative analysis	United Kingdom	47 Patients	Patients only	Not reported	Fear Communication/information, Continuity of care, Financial issues, Lifestyle changes, Social support, Understanding of treatments and devices Awareness of dying, Hope, Spiritual support	
Gonzalez-Jaramillo V et al. [60]	Unmet Needs in Patients With Heart Failure: The Importance of Palliative Care in a Heart Failure Clinic	Secondary qualitative analysis	Switzerland	31 Patients	Patients only	Mixed (HFrEF 58%, HFmrEF 26%, HFpEF 16%)	Fear,, Referral to palliative care Advance care planning, Communication/information, Continuity of care, Sense of isolation, Understanding prognosis, Understanding of treatments and devices Hope, Spiritual support	
Harding R et al. [61]	Meeting the Communication and Information Needs of Chronic Heart Failure Patients	Qualitative study	United Kingdom	20 Patients 11 Caregivers	Both (not paired)	Predominantly HFrEF (EF 22.5%−50, mean 34% (SD 8.33))	Oedema burden, Anger, Anxiety, Cognitive Impairment, Depression, Guilt, Frustration Advance care planning, Communication/information, Understanding of treatments and devices	Limited or lack of communication with HCPs, Understanding of treatments and devices
Mahoney-Davies et al. [62]	Examining the emotional and psychological experiences of people with heart failure	Qualitative mixed methods study	United Kingdom	10 Patients	Patients only	Not reported	Fatigue, Sleep disorders Anger, Depression, Fear, Frustration, Worries about health Being connected, Communication/information, Changes in dyad roles, Continuity of care, Family concerns, Financial issues, Lifestyle changes, Understanding of treatments and devices Awareness of dying, Being autonomous or in control, Illness perception, Hope, Need to cope with the disease	
McIlfatrick S et al. [63]	‘The importance of planning for the future’: Burden and unmet needs of caregivers’ in advanced heart failure: A mixed methods study	Qualitative mixed methods study	Ireland, United Kingdom	112 Patients 84 Caregivers	Both (not paired)	HFrEF (Ejection fraction ⩽40%)	Anxiety, Depression	Physical health Anxiety, Depression, Emotional strain/burden Care continuity, Changes in dyad roles/family issues, Limited or lack of communication with HCPs, Social support, Understanding of Palliative Care Limited/inadequate end-of-life and bereavement support, Unpreparedness/fear for the future
Murray S A et al. [64]	Patterns of Social, Psychological, and Spiritual Decline Toward the End of Life in Lung Cancer and Heart Failure	Qualitative study	United Kingdom	24 Patients	Patients only	Not reported	Anxiety, Depression, Frustration, Family concerns, Sense of isolation Awareness of dying, Being autonomous or in control, Having meaning, Hope, Religiosity, Spiritual support	
Nordfonn OK et al. [65]	Patients’ experience with heart failure treatment and self-care—A qualitative study exploring the burden of treatment	Qualitative study	Norway	17 Patients	Patients only	Not reported	Anxiety, Worries about health, Guilt Continuity of care, Communication/information, Family concerns, Lifestyle changes, Sense of isolation, Understanding of treatments and devices Being autonomous or in control, Illness perception, Need to cope with the disease	
Nordgren L et al. [66]	Living With Moderate-Severe Chronic Heart Failure as a Middle-Aged Person	Qualitative study	Sweden	7 Patients	Patients only	Not reported	Anxiety, Self-esteem, Guilt, Frustration Being connected, Lifestyle changes, Family concerns, Understanding of treatments and devices Awareness of dying, Being autonomous or in control, Illness perception, Having meaning, Hope, Dignity, Need to cope with the disease	
Oriani A et al. [67]	What are the main symptoms and concerns reported by patients with advanced chronic heart failure?—a secondary analysis of the Palliative care Outcome Scale (POS) and Integrated Palliative care Outcome Scale (IPOS)	Secondary quantitative and qualitative analysis	SwitzerlandUnited Kingdom	102 Patients	Patients only	40 patients with HFpEF or HFrEF, 62 patients not reported	Altered bowel, Cough, Breathlessness/dyspnoea, Dizziness/drowsiness, Fatigue, Oedema burden, Pain, Poor appetite, Poor mobility, Pruritus, Sleep disorders Anxiety, Cognitive Impairment, Self-esteem, Concerns about comorbidities, Depression, Sense of isolation, Family concerns, Understanding of treatments and devices	
Pattenden JF et al. [68]	Living with heart failure; patient and carer perspectives	Qualitative study	United Kingdom	36 Patients 36 Caregivers	True dyadic (paired)	Not reported	Altered bowel, Breathlessness/dyspnea, Fatigue, Dizziness/drowsiness, Falls, Pain, Oedema Burden, Sleep disorders Anger, Anxiety, Cognitive impairment, Concerns about comorbidities, Depression,, Fear, Frustration Changes in dyad roles, Communication/information, Financial issues, Lifestyle changes, Sense of isolation, Social support, Understanding prognosis, Understanding of treatments and devices Awareness of dying, Being autonomous or in control, Having meaning, Hope, Illness perception, Religiosity, Spiritual Support	Physical burden of caring 24/7 Anxiety, Emotional strain burden Social and financial burden of care, Social support, Understanding of treatments and devices
Paturzo M et al. [69]	The lived experience of adults with heart failure: a phenomenological study	Qualitative study	Italy	30 Patients	Patients only	Not reported	Anger, Frustration, Worries about health, Being connected, Lifestyle changes, Family concerns, Financial issues, Sense of isolation Awareness of dying, Being autonomous or in control, Having meaning, Hope, Illness perception, Need to cope with the disease, Religiosity, Unpreparedness/fear for the future	
Pihl E et al. [70]	Depression and health-related quality of life in elderly patients suffering from heart failure and their spouses: a comparative study	Quantitative study	Sweden	47 Patients 47 Caregivers	True dyadic (paired)	Not reported	Depression	Physical health Depression, Emotional strain/burden
Romanò M et al. [71]	Palliative Care for Patients with End-Stage, Non-Oncologic Diseases—A Retrospective Study in Three Public Palliative Care Departments in Northern Italy	Quantitative study	Italy	55 Patients	Patients only	Not reported	Breathlessness/dyspnoea, Fatigue, Pain	
Ross L et al. [72]	Spiritual needs and spiritual support preferences of people with end-stage heart failure and their carers: implications for nurse managers	Qualitative interview	United Kingdom	16 Patients	Patients only	Not reported	Being connected, Being autonomous or in control, Having meaning, Hope	
Ryan M et al. [73]	Living with an unfixable heart: A qualitative study exploring the experience of living with advanced heart failure	Qualitative study	Ireland	9 Patients	Patients only	Not reported	Fatigue Anxiety, Fear, Depression, Frustration, Guilt Communication/information, Continuity of care, Family concerns, Lifestyle changes, Sense of isolation Being autonomous or in control, Hope, Need to cope with the disease, Dignity	
Small N et al. [74]	Dying, death and bereavement: a qualitative study of the views of carers of people with heart failure in the UK	Qualitative study	United Kingdom	20 Caregivers (reporting also on dyads)	Bereaved Caregivers only (reporting also on dyads)	Not reported	Advance care planning, Communication/information Religiosity, Spiritual support	Emotional strain/burden Limited or lack of communication with HCPs, Social isolation, Social support Limited/inadequate end-of-life and bereavement support, Preparing for/talking about death
Stocker R et al. [75]	Should heart failure be regarded as a terminal illness requiring palliative care? A study of heart failure patients’, carers’ and clinicians’ understanding of heart failure prognosis and its management	Qualitative study	United Kingdom	13 Patients 9 Caregivers	Both (not paired)	Not reported	Anxiety, Referral to Palliative Care Communication/information, Understanding prognosis Unpreparedness/fear for the future	Limited or lack of communication with HCP
Walsh M et al. [76]	Heart failure symptom burden in outpatient cardiology: observational cohort study	Quantitative study	Ireland	22 Patients	Patients only	Not reported	Altered bowel, Breathlessness/dyspnoea, Muscle weakness, Oedema burden, Pain, Poor mobility Anxiety, Depression, Worries about health Communication/information, Family concerns	
Walsh M et al. [77]	Patients with Congestive Cardiac Failure Referred to Specialist Palliative Care	Quantitative study	Ireland	57 Patients	Patients only	Not reported	Cough, Breathlessness/dyspnoea, Fatigue, Oedema burden End-of-life care	
Walthall H et al. [78]	Living with breathlessness in chronic heart failure: a qualitative study	Qualitative study	United Kingdom	25 Patients	Patients only	HFrEF	Breathlessness/dyspnoea, Cough, Sleep disorders Anxiety, Depression, Fear, Frustration, Emotional Distress Changes in dyad roles, Lifestyle changes, Sense of isolation, Understanding of treatments and devices Awareness of dying, Being autonomous or in control, Illness perception, Having meaning, Need to cope with the disease	
Walthall H et al. [79]	Patients experience of fatigue in advanced heart failure	Qualitative study	United Kingdom	23 Patients	Patients only	HFrEF	Fatigue, Sleep disorders Depression, Frustration Lifestyle changes, Sense of isolation Being autonomous or in control, Need to cope with the disease, Unpreparedness/fear for the future	
Younas A et al. [80]	Perceived Social Support and Associated Factors among Caregivers of Individuals with Heart Failure: A Convergent Mixed Methods Study	Mixed methods study	Italy, Spain, The Netherlands	158 Caregivers	Caregivers only	Not reported		Emotional strain/burden Social support, Changes in dyad roles/family issues

These studies were conducted across nine countries, with the United Kingdom being the most represented (18 papers), followed by Sweden (6 papers) and Italy (5 papers). Additional contributions came from Spain, the Netherlands, Ireland, Switzerland, Norway, and one paper combining Switzerland and the United Kingdom.

On 34 retrieved papers, 3 reported the paired dyadic perspective, 5 reported unpaired patients and caregivers experiences, 20 only patients’ and 6 only caregivers’ experience (of these, one indirectly recalling the dyadic experience, see Table 1). Disease severity was heterogeneously described across included studies and the mapped needs reflect experiences across varying stages of chronic HF. Supplement 3 reports detailed symptoms and concerns extracted from retrieved papers.

**Fig. 2:**
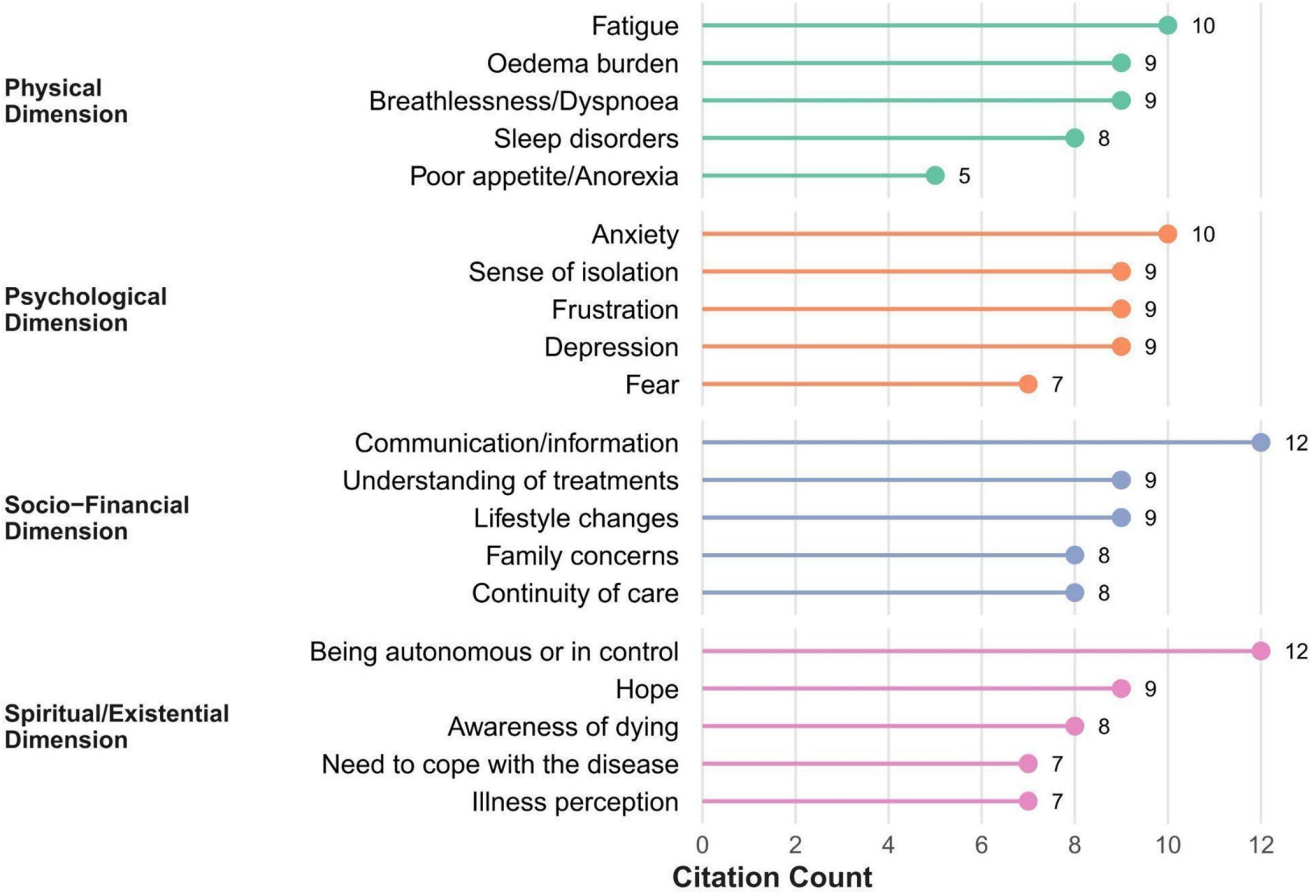
Most cited needs and concerns across four dimensions (Physical, Psychological, Socio-Financial, Existential/Spiritual) for patients. Citation counts, represented by the length of lollipop segments, indicate the number of papers citing the specific need/concern. Dimensions are shown on the left, with corresponding needs/concerns labelled adjacent to each lollipop segment. Citation counts reflect reporting frequency in the literature and do not represent clinical prevalence

**Fig. 3:**
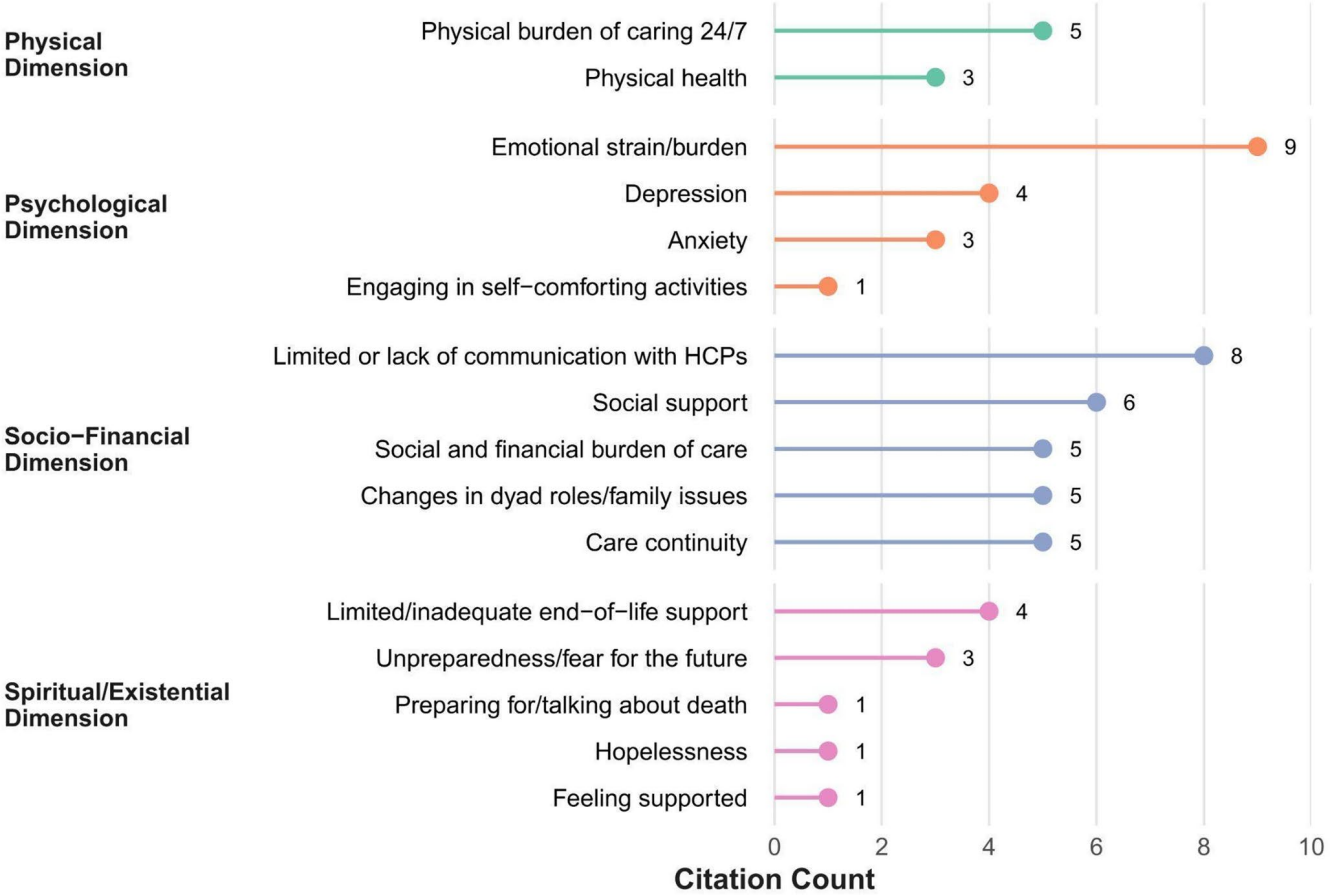
Most cited needs and concerns across four dimensions (Physical, Psychological, Socio-Financial, Existential/Spiritual) for caregivers. Citation counts, represented by the length of lollipop segments, indicate the number of papers citing the specific need/concern. Dimensions are shown on the left, with corresponding needs/concerns labelled adjacent to each lollipop segment. Citation counts reflect reporting frequency in the literature and do not represent clinical prevalence

Citation frequency reflected reporting patterns in the literature and should not be interpreted as a direct measure of clinical prevalence or importance; less frequently reported issues, particularly in the existential/spiritual domain, may be under-detected despite being relevant to the dyads. Figures 2 and 3 report needs within each domain.

### Heart failure phenotype

Ejection fraction or heart failure phenotype (HFrEF, HFmrEF, HFpEF) was reported in 7 of the 34 included studies [57, 60, 61, 63, 67, 78, 79], see Table 1. In these studies, patients predominantly had reduced ejection fraction (HFrEF or EF ≤ 40%), with one study reporting a mixed phenotype distribution (HFrEF 58%, HFmrEF 26%, HFpEF 16%) [60]. None of the included studies reported needs stratified by ejection fraction and, as such, a phenotype-specific analysis could not be performed.

### Dyad-level synthesis

Most studies examined patients or caregivers separately and only 8 studies included both perspectives. None employed formal interdependence models or dyadic analyses; therefore, our findings reflect co-occurrence of reported needs rather than established directionality or magnitude [48, 50, 51, 61, 63, 68, 70, 75]. However, the thematic synthesis identified dyad-level mechanisms encompassing the physical, psychological, social, and existential dimensions. Some mechanisms extended across multiple domains (e.g., social and existential).

### Shared prognostic and illness-related uncertainty

The unpredictable trajectory of chronic HF generated shared prognostic and illness-related uncertainty in the dyads. Fluctuating patients’s symptoms, including breathlessness, fatigue, and oedema, co-occurred with high levels of anxiety and depression in both patients and caregivers [47, 48, 52, 54, 56, 62–68, 70, 71, 73, 75–79]. Recurrent communication needs regarding prognosis, treatment, device management, and ACP further reflected ongoing efforts to manage uncertainty [47, 48, 50–52, 55, 58–62, 65–68, 73–76, 78]. Existential concerns, including awareness of dying and fears about the future, extended beyond clinical instability [50, 53, 55, 59, 62–64, 66, 68, 69, 74, 78].

### Role renegotiation within the dyad

Progressive functional decline and dependency of patients were associated with reconfiguration of the dyads. Patients reported muscle weakness and cognitive impairment, alongside concerns regarding autonomy and control; caregivers reported physical exhaustion, health deterioration, and emotional burden [48, 49, 53, 54, 57, 63, 68, 70, 74, 80]. Explicit changes in dyadic roles were described, together with family-related concerns [47, 48, 50, 52, 55, 56, 58, 61, 62, 64–69, 72, 73, 76, 78, 79].

### Healthcare system navigation as a dyadic burden

Navigating the healthcare systems emerged as a shared burden. Concerns regarding continuity of care, communication gaps with professionals, and informational needs related to treatments and device management were frequently described [47, 48, 50–55, 57–63, 65–68, 73–75, 78]. Caregivers additionally highlighted care coordination challenges and limited access to supportive or palliative services [49–51, 54, 57, 63]. Financial toxicity and unmet social needs in both patients and caregivers further contributed to this shared burden [53, 54, 59, 62, 63, 68, 69, 74, 80].

### Dyadic existential adaptation

Beyond symptoms and care-related burdens, patients and caregivers described existential concerns related to dignity and autonomy, maintenance of hope, and meaning-making in the context of progressive illness, including spiritual or religious dimensions [47, 48, 50, 52, 55, 59, 60, 62, 64–66, 68, 69, 72–74, 78, 79]. Caregivers reported existential distress, hopelessness, and difficulties preparing for death or accessing bereavement support [51, 54, 57, 63, 74].

## Discussion

Although dyadic interdependence has been demonstrated in prior research [81–83], to the best of our knowledge, this is the first scoping review to specifically examine the multidimensional needs and concerns of both patients with chronic HF and their IC in European countries from a dyadic perspective.

Most included studies were qualitative or descriptive and were not designed to formally test dyadic directionality (e.g., actor–partner models). However, results showed consistent co-occurrence and perceived reciprocity of needs across patients and caregivers.

The dyadic interpretation presented here should be understood as a conceptual synthesis grounded in reported experiences. However further studies are needed to explore directionality.

### Physical dimension

Patients with chronic HF experience a substantial physical symptom burden, most commonly breathlessness, fatigue, and oedema, which significantly impair QoL and daily functioning [47, 48, 52, 56, 67, 68, 71, 76–78]. Sleep disturbances, pain, and muscle weakness further worsens this burden [48, 52, 56, 62, 67, 68, 71, 76, 78, 79]. Symptom clusters often fluctuate unpredictably, reinforcing functional dependence and limiting autonomy. As clinical status deteriorates, patients’ dependency increases, contributing to physical exhaustion and health deterioration among caregivers. Additionally, the increasing reliance on caregivers for symptom monitoring, medication management, and basic daily activities, often culminates in unplanned hospital admissions or, in advanced stages, institutionalisation, further disrupting dyadic balance and accelerating role transitions for both patient and caregiver [49, 53, 54, 57, 63, 68, 70].

### Psychological dimension

Anxiety, depression, and emotional distress are prevalent among patients and caregivers, though they manifest differently. Patients frequently experience fear, guilt, and frustration linked to loss of control over their condition, with severity correlating with functional loss and physical symptom burden [48, 52, 56, 59–70, 73, 75, 76, 78, 79]. Caregivers report depression and anxiety driven by the unpredictable disease trajectory, caregiving demands, and persistent uncertainty about prognosis [48, 49, 53, 54, 57, 63, 68, 70, 74, 80]. Limited prognostic communication with healthcare professionals further compounds this shared emotional burden, reinforcing the need for psychological support for both the dyad’s members [50, 51, 53, 54, 57, 61, 63, 74, 75].

### Socio-financial dimension

Social isolation represents a significant and underrecognised burden in chronic HF, compounded by functional limitations, medication side effects (e.g. diuretics), and, in advanced stages, oxygen dependency [59, 62, 65, 66, 68, 69, 73, 78, 79, 84]. For older patients, isolation is further intensified by the concurrent loss of social networks [52]. Reduced social engagement frequently exacerbates psychological burden and undermines treatment adherence [67, 85].

Financial burden is related to treatment costs, care expenses, and in younger patients, premature career disruption. Inadequate financial and social support significantly increases out-of-pocket costs [49–51, 54, 57, 59, 62, 63, 66, 68, 69, 86, 87].

### Existential/spiritual dimension

As patients recognise chronic HF as a life-limiting condition, needs around meaning, hope, and dignity become prominent throughout the illness trajectory. Social isolation may amplify existential distress, impairing patients’ sense of control and capacity for advance planning, and potentially contributing to unplanned admissions and loss of dignity at end of life [47, 48, 50, 52, 55, 59, 60, 62, 64–66, 68, 69, 72, 73, 77–79]. Caregivers face a parallel existential burden, marked by hopelessness, anticipatory grief, and unmet needs for spiritual support as they accompany their loved ones toward death [49, 53, 54, 63, 80].

Together, these domain-specific findings suggest that the dyadic experience of chronic HF is structured by shared relational processes rather than isolated burdens.

### Dyad-level propositions for early palliative care integration

**Fig. 4:**
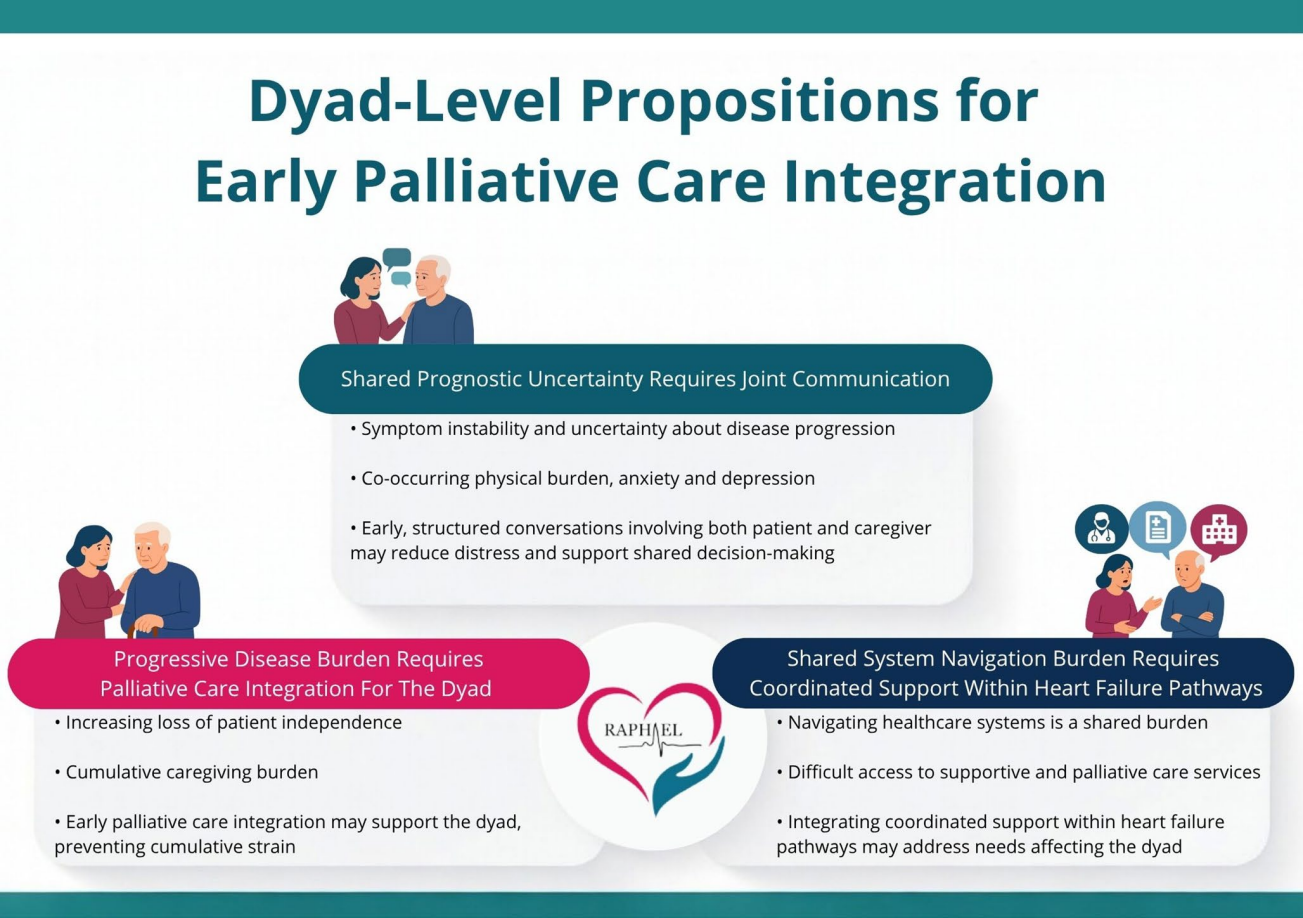
Dyadic model of multidimensional needs in chronic HF, highlighting three modifiable interfaces for early palliative care intervention

Synthesising the meta-aggregated findings across the included studies, three dyad-level propositions emerge that identify potentially modifiable targets for intervention (see Fig. 4).

### Shared prognostic uncertainty requires joint communication

Across studies, both patients and caregivers reported distress linked to symptom instability and uncertainty about disease progression [48, 68, 70], together with unmet needs for clearer information, prognostic discussions, and ACP [50, 51, 60, 61, 63, 75]. Physical symptom burden frequently co-occurred with anxiety and depression in both members of the dyad [47, 48, 52–54, 56, 61–68, 70, 71, 73, 75–79]. Taken together, these findings indicate that uncertainty about prognosis and future care represents a shared challenge within the dyad. Early, structured conversations involving both patient and caregiver may therefore reduce distress and support shared decision-making.

### Disease progression and caregiving burden

Paired and dyadic studies documented increasing caregiver responsibilities alongside patient loss of independence [48, 50, 61, 63, 68, 70, 75]. Patients expressed concerns about autonomy and control, while caregivers reported physical exhaustion, emotional burden, and health decline [47–50, 52–55, 57, 62–66, 68–70, 72, 73, 78–80]. This convergence suggests that progressive disease brings cumulative, shared burden. Early palliative care integration may support both patients and caregivers, preventing dyadic strain.

### Fragmented care and shared system navigation burden

Patients and caregivers consistently described challenges related to continuity of care, as well as difficulties accessing supportive and palliative services [47–51, 53, 54, 57–61, 63, 65, 73–75]. Financial strain and unmet social support needs further affected the dyad [53, 54, 59, 62, 63, 68, 69, 74, 80]. These findings suggest that navigating the healthcare system constitutes a shared workload rather than an individual burden. Integrating coordinated support within heart failure pathways, such as a designated contact person or care coordinator, may address needs affecting both patient and caregiver.

### Why early PC involvement is important in patients with chronic HF

The bidirectional relationship between the physical health of patients with chronic HF and that of their IC highlights the need for a comprehensive, dyadic approach to disease management, including early PC referral and robust social support.

As recently mapped by Pastrana et al., definitions of “advanced” or “symptomatic” HF and recommended triggers for palliative care referral vary substantially across Europe [89]. This heterogeneity further supports a needs-based rather than stage-based approach to palliative care integration.

Prognostication in chronic HF remains challenging; persistent and often refractory symptoms frequently lead to recurrent hospitalisations, which commonly act as triggers for late, and sometimes very late, palliative care referral [2, 26, 28, 40]. This approach risks leaving earlier sources of suffering unaddressed, including non-physical concerns that may become burdensome if not managed proactively [10, 40, 57, 81]. This underscores the importance of a structured approach to integrated palliative care involvement, as recommended by European and US guidelines [1, 84].

This integration becomes particularly critical as patients approach the end-of-life phase, when limited or inadequate care and bereavement support concern some caregivers [51, 57, 63, 74].

As in other life-limiting conditions, PC team involvement with the inclusion of a spiritual care provider, may deliver important existential support and add overall quality to care [88]. When home PC teams have supported the patients in their last phase, bereaved caregivers often express significant satisfaction in facilitating their loved one’s death at home, a positive outcome that outweighs any challenges they have faced [57].

Despite these demonstrated benefits, many patients recognize the potential value of PC yet remain confused about its role in chronic HF or reluctant to discuss it due to its perceived association with end-of-life care and dying [50, 51, 60, 75].

Many patients report ACP discussion as an important unmet need that should be fulfilled by the caring team [48, 50, 51, 60, 61, 74]. The need for ACP may itself function as a trigger for palliative care involvement, as these discussions encompass transitions to home care, end-of-life decisions, and other non-medical issues requiring a multidisciplinary approach.

Finally, advanced device therapies (such as implantable cardioverter-defibrillators, cardiac resynchronisation therapy devices, or left ventricular assist devices) introduce additional complexity for the dyad, including device management, shock-related anxiety, and decisions regarding deactivation at end-of-life [50, 51, 57, 60, 61, 63, 68, 75]. At the same time, emerging evidence suggests that device-based remote multiparameter monitoring may support earlier detection of clinical deterioration, reduce hospitalisations, and indirectly alleviate caregiver burden [90, 91]. These technologies may therefore function not only as therapeutic interventions but also as organisational and supportive tools. In this context, early integration of palliative care may facilitate ACP around both implantation and deactivation, aligning technological interventions with patient and caregiver goals.

## Limitations

This review did not include a formal methodological quality appraisal, consistent with scoping review methodology. However, characteristics of the included studies may influence how reported needs are represented in the literature. Many studies recruited participants from specialist or tertiary heart failure clinics, which may preferentially reflect the experiences of patients with advanced disease or established access to specialist services. Several qualitative studies were based on small purposive samples. Studies involving bereaved caregivers may be subject to recall and retrospective reinterpretation bias. In addition, certain populations appear underrepresented in the literature, including individuals with HFpEF, patients with cardiac devices, and socioeconomically disadvantaged groups.

There is paucity of peer-reviewed literature specifically addressing the non-physical needs of patients with chronic HF, and even less focusing on the multidimensional needs of their caregivers. Studies exploring non-oncological advanced disease often include heterogeneous samples (e.g., chronic obstructive pulmonary disease, cardiorenal syndromes), with only a small proportion of patients with chronic HF. Furthermore, the exclusion of non-European studies (e.g., US, Australia, Japan, China) limits generalisability. However, as many palliative care needs are culturally shaped, this restriction is consistent with the aim of the present review, which focused on the European context.

Additionally, the included studies showed a geographic imbalance, with the United Kingdom contributing 18 of the 34 studies, followed by Sweden and Italy. As a result, the findings may reflect characteristics of healthcare systems where palliative care integration and dyadic research are more established. This should be considered when interpreting the broader European applicability of the results.

## Conclusions

This scoping review maps the multidimensional needs of European adults living with chronic HF and their informal caregivers across the disease trajectory from a dyadic perspective. Patients and caregivers experience substantial physical, psychological, socio-financial, and existential burdens. Although most included studies did not formally model dyadic interdependence, consistent co-occurrence of needs supports understanding chronic HF as a relational experience.

These findings call for a needs-based approach and strengthen the rationale for earlier, structured integration of palliative care within HF pathways. Future research should adopt robust dyadic methodologies and develop strategies to proactively identify and address shared sources of distress.

Tables.

## Supplementary information

Below is the link to the electronic supplementary material.

Supplementary Material 1 (PDF 74.4 KB)

Supplementary Material 2 (PDF 74.4 KB)

Supplementary Material 3 (PDF 69.8 KB)

## Data Availability

No datasets were generated or analysed during the current study.
