# Supplementary material for: The multidimensional needs of chronic heart failure patients and caregivers from a dyadic perspective: a scoping review
Source: Heart Fail Rev. 2026 Mar 27;31(1):45. doi: 10.1007/s10741-026-10616-4 (PMC13031253; doi:10.1007/s10741-026-10616-4)
Supplement: Supplementary file 1 — Supplementary Material 1 (PDF 74.4 KB) [file 10741_2026_10616_MOESM1_ESM.pdf]

The Multidimensional Needs of Chronic Heart Failure Patients and Caregivers from a dyadic perspective: A Scoping Review

Authors

Prof. Dr. Cosimo Chelazzi[1,2] 0000-0002-5156-8638

Dr. Daniele Marelli[1] 0009-0005-1527-2118

Prof. Dr. Carla Ida Ripamonti[1] 0000-0001-5495-5054

Dr. Matteo Pagnesi[3] 0000-0002-9298-7871

Prof. Dr. Marco Metra[4] 0000-0001-6691-8568

Prof. Dr. Philip Larkin[5] 0000-0001-8424-3920

Prof. Dr. Geert-Jan Geersing[6] 0000-0001-6976-9844

Dr. Carlo Leget[7] 0000-0002-6647-8141

Dr. Klaus Witte[8] 0000-0002-7146-7105

Dr. Everlien de Graaf[6] 0000-0001-8528-4070

on behalf of the RAPHAEL Consortium.

1. Department of Medical and Surgical Specialties, Radiological Sciences, and Public Health, Università degli Studi di Brescia, Brescia, Italy
2. SC Cure Palliative e ADI, ASST Spedali Civili di Brescia, Brescia, Italy
3. Institute of Cardiology, ASST Spedali Civili, University of Brescia, Brescia, Italy
4. Cardiology, Vita-Salute San Raffaele University; IRCCS San Raffaele Hospital. Milan. Italy
5. Lausanne University Hospital and University of Lausanne, Lausanne, Switzerland.
6. Universiteit Medisch Centrum Utrecht, Julius Center for Health Sciences and Primary Care – Dept. of General Practice and Nursing Science, Utrecht University, Utrecht, The Netherlands
7. Department of Care Ethics, University for Humanistic Studies, Utrecht, The Netherlands
8. University of Leeds, Leeds, UK

Co-corresponding Authors:

Matteo Pagnesi, Institute of Cardiology, ASST Spedali Civili, University of Brescia, Brescia, Italy, m.pagnesi@gmail.com

Cosimo Chelazzi, Department of Medical and Surgical Specialties, Radiological Sciences, and Public Health, Università degli Studi di Brescia, Brescia, Italy; SC Cure Palliative e ADI, ASST Spedali Civili di Brescia, Brescia, Italy, cosimo.chelazzi@unibs.it

Supplementary material 2- Search strategy for MEDLINE

(("Heart Failure"[Mesh] OR "Heart Failure"[tiab:~2]) AND (Advanced[tw] OR Chronic[tw]))

AND

(("Caregivers"[Mesh] OR Caregiver*[tw] OR "Care Giver*"[tw] OR "Care Givers"[tiab:~2]) OR ("Patients"[Mesh] OR Patient*[tw]))

AND

(("Spirituality"[Mesh] OR Spirituality[tw] OR "Inner need*"[tw] OR "Spiritual support*"[tw] OR "Spiritual role"[tw] OR "Spiritual need*"[tw] OR "Religious support"[tw] OR "Religious role"[tw] OR "Religious help"[tw]) OR "Social need"[tiab:~2] OR "Social concern"[tiab:~2] OR "Care giver needs"[tiab:~2] OR "Caregiver needs"[tiab:~2] OR "Meaning making"[tiab:~2] OR "Palliative Care"[majr] OR "Psychological symptoms"[tiab:~2] OR "Physical symptoms"[tiab:~2] OR Loneliness[tw] OR (Existential[tw] AND (Need*[tw] OR Concern*[tw] OR Issue*[tw])) OR ("Financial Stress"[MeSH Terms] OR "financial toxicit*"[tw] OR "financial pressure*"[tw] OR "financial hardship*"[tw] OR "economic hardship*"[tw]))

AND

(2000:2024[pdat])
