# Supplementary material for: The multidimensional needs of chronic heart failure patients and caregivers from a dyadic perspective: a scoping review
Source: Heart Fail Rev. 2026 Mar 27;31(1):45. doi: 10.1007/s10741-026-10616-4 (PMC13031253; doi:10.1007/s10741-026-10616-4)
Supplement: Supplementary file 2 — Supplementary Material 2 (PDF 74.4 KB) [file 10741_2026_10616_MOESM2_ESM.pdf]

Physical dimension

According to the literature patients with HF consistently reported a wide range of physical symptoms that significantly impaired their QoL. The most common symptoms were breathlessness/dyspnoea [47, 48, 52, 56, 67, 68, 71, 76-78], followed by fatigue [47, 48, 52, 56, 62, 67, 68, 71, 73, 77, 79] and oedema burden [48, 52, 56, 58, 61, 67, 68, 76, 77]. Other frequently reported symptoms included pain [56, 67, 68, 71, 76], sleep disturbances [48, 52, 56, 62, 67, 68, 78, 79], and muscle weakness [48, 56, 76]. Less commonly, patients described mouth problems and thirst [56]. Caregivers commonly experienced physical exhaustion and fatigue due to the intense, round-the-clock demands of caregiving [49, 53, 54, 57, 68]. Many expressed concerns about deterioration in their own physical health [54, 63, 70] and its impact on their ability to provide sustained care.

Psychological dimension

The psychological burden of HF was profound. Anxiety [48, 52, 56, 61, 63-68, 73, 75, 76, 78] and depression [48, 56, 61-64, 67, 68, 70, 73, 76, 78, 79] were the most prevalent psychological symptoms. Patients commonly described frustration [48, 52, 61, 62, 64, 66, 68, 69, 73, 78, 79], fear [52, 59, 60, 62, 68, 73, 78], and anger [61, 62, 68, 69]. Additional concerns included guilt [61, 65, 66, 73], worries about common comorbidities (i.e. renal and pulmonary) [58, 67, 68] and broader concerns regarding overall health-status [62, 65, 69, 76]. Cognitive impairment, memory deficits [61, 67, 68], low self-esteem [52, 66, 67], and general emotional distress [78]. Caregivers faced significant psychological distress, with high prevalence of anxiety [54, 63, 68], depression [53, 54, 63, 70], and overall emotional burden [48, 49, 53, 54, 57, 63, 68, 70, 74, 80]. Some adopted coping strategies such as exercise or hobbies to mitigate emotional stress [54]. Key unmet communication needs included the desire for clear, reliable information about their condition [47, 48, 50-52, 58-62, 68, 73-76], support with advance care planning [48, 50, 51, 60, 61, 74], better understanding of treatments and device management [50-52, 55, 59-62, 65-68, 78], and prognostic clarity [52, 60, 68].

Social and financial dimension

Patients reported substantial social and financial challenges. Social isolation [48, 52, 60, 64, 65, 67-69, 73, 78, 79], the need for major lifestyle adjustments [59, 62, 65, 66, 68, 69, 73, 78, 79], and concerns about continuity of care [47, 48, 50, 55, 58-60, 62, 65, 73] were often reported as major issues. Additional concerns included difficulty maintaining social connections [50, 55, 58, 62, 66, 69, 72], family-related concerns [58, 62, 64-67, 69, 73, 76], and changes in dyadic roles [62, 68, 78]. Financial toxicity, driven by prolonged illness, loss of income, and increased expenses,was also frequently reported [59, 62, 68, 69], together with broader unmet needs for social support [59, 68]. IC' socio-financial burdens frequently mirrored those reported by patients [48, 50, 54, 68]. Caregivers frequently highlighted challenges in coordinating continuity of care [49, 50, 51, 54, 57, 63], exacerbated by inadequate communication with healthcare providers [50, 51, 53, 54, 57, 61, 63, 74, 75] and limited access to supportive services [50, 57]. Key information gaps concerned treatments and device management [50, 51, 57, 61, 68], and PC options [57, 63]. Additional burdens included changes in dyadic roles [48, 53, 54, 63, 80], social isolation [48, 49, 53, 74], and unmet needs for social support [53, 54, 63, 68, 74, 80].

Existential/spiritual dimension

Existential/spiritual concerns of patients were prominent, particularly regarding the awareness of dying [50, 55, 59, 62, 64, 66, 68, 69, 78], alongside the need for high-quality end-of-life care [48, 77]. Preserving autonomy and control emerged as a central concern of patients [47, 48, 50, 52, 55, 62, 64-66, 68, 69, 72, 73, 78, 79]. Patients frequently expressed the need to find meaning in their illness [48, 55, 64, 66, 68, 69, 72, 78] and to maintain hope [48, 59, 60, 62, 64, 66, 68, 69, 72, 73]. Illness perceptions [55, 62, 65, 66, 68, 69, 78], coping with the disease [62, 65, 66, 69, 73, 78, 79] were also frequently noted. Some patients preferred limited information to avoid distress, while others expressed concerns about being unprepared or fears about the future [69, 75, 79]. Dignity [66, 73], religiosity [64, 68, 69, 74], and the desire for spiritual support [52, 59, 60, 64, 68, 74] also emerged as important themes. Existential and spiritual distress was common among caregivers. While some felt supported [49], many reported hopelessness [54] and inadequate end-of-life or bereavement support [51, 57, 63, 74]. Difficulties in preparing for or discussing death of their loved one [74], along with pervasive feelings of unpreparedness and fears about the future [53, 63] significantly impaired caregiver QoL, particularly during the terminal phase.
