# Supplementary material for: The multidimensional needs of chronic heart failure patients and caregivers from a dyadic perspective: a scoping review
Source: Heart Fail Rev. 2026 Mar 27;31(1):45. doi: 10.1007/s10741-026-10616-4 (PMC13031253; doi:10.1007/s10741-026-10616-4)
Supplement: Supplementary file 3 — Supplementary Material 3 (PDF 69.8 KB) [file 10741_2026_10616_MOESM3_ESM.pdf]

Supplementary material 1 - Detailed methodology

Stage 1: Identifying the Research Question(s)

The main research question was to determine the multidimensional needs of patients with chronic HF and their caregivers through the existing peer-reviewed international literature. Evidence was sought regarding the following questions:

1. What are the main physical symptoms reported by patients living with chronic HF?
2. What are the psychological needs of patients with chronic HF and their caregivers?
3. What are the social and financial needs of patients with chronic HF and their caregivers?
4. What are the spiritual needs of patients with chronic HF and their caregivers?

Stage 2: Identifying Relevant Studies

Relevant studies were identified using the Participants, Concept, Context (PCC) framework.

**Participants/Population:** We considered adult populations with the diagnosis of chronic HF, and their informal caregivers. All types and definitions of chronic HF due to all etiologies with symptoms and needs described above were included.

**Concept:** The concept of interest for this scoping review was to search through the literature for studies on the multidimensional needs of patients with chronic HF and those of their caregivers.

**Context:** All European countries were considered. Literature from other countries was considered only if it included clear and separable data from European patients.

**Inclusion Criteria:** Studies/documents were included if they satisfied the following criteria:

- All adult patients with chronic HF.
- Reporting directly on the multidimensional needs of patients with chronic HF and those of their caregivers.
- Year of publication: between January 2000 and January 2024.
- Language: English.
- Geographic contest: Europe.

**Exclusion Criteria:** Anything that did not meet the inclusion criteria was excluded.

Stage 3: Study Selection

**Search Strategy:** The search strategy included terms describing the population (adults with chronic HF) and the concept of interest (physical symptoms, psychological symptoms, existential/spiritual, social/financial need; patients and caregivers).

A preliminary search was done on MEDLINE by DM, CR and CC to find relevant keywords. Boolean operators were used to refine search terms for MEDLINE (Supplement 1) and then also adapted for the following electronic bibliographic databases: EMBASE, PsycINFO, CINAHL, The Cochrane Library (Cochrane Database of Systematic Reviews, Cochrane Central Register of Controlled Trials (CENTRAL) Database, and Web of Science (science and social science citation index). Reference

lists of relevant reviews and key articles were hand-checked. Grey literature, editorials, and letters to the editor were also excluded.

Stage 4: Charting the Data

Retrieved abstracts were uploaded on the RAYYAN tool for deduplication, screening, and analysis.[43]

The abstract screening was carried out independently and blindly by CC and CR. Any conflicts were resolved through team discussion until a consensus was reached. If there were persistent conflicts or doubts, a third researcher (DM) was consulted.

The selected full texts were then reviewed independently and blindly by CR and CC for inclusion. Any discrepancies were resolved through team discussion until a consensus was reached. If there were doubts or conflicts regarding the selection of full papers for analysis, a third researcher (DM) was involved. Exclusion reasons were noted and documented in the PRISMA-ScR diagram within the final report (see Fig. 1).

Finally, data about the population, concept, and context outlined in the review question, along with any other pertinent details, were extracted and summarised.

Data extraction for each document/article was performed by three investigators (CR, CC, DM) and a quality check was independently done by EvG. Any discrepancies that arose were addressed through team discussion until a consensus was reached. No additional or supplementary information was required to determine eligibility, so there was no need to contact the authors of the articles.

**Data Charted:**

1. First Author
2. Title
3. Design/methodology of the study
4. The country where the study was published or conducted
5. Sample size
6. Patient’s symptoms and needs and concerns
7. Caregivers symptoms, needs and concerns

Stage 5: Collecting, Summarizing, and Reporting the Results

The data were used to assess the multidimensional needs reported by patients with chronic HF, including, if reported, their frequency and intensity. The needs of caregivers were also assessed.

Stage 6: Consultation and Conclusions

The team drew conclusions after all the data had been analysed. The results were shared with other Consortium members, including patient stakeholders and healthcare professionals.
